# Histone Deacetylase Inhibition: An Important Mechanism in the Treatment of Lymphoma

**DOI:** 10.3969/j.issn.2095-3941.2012.02.001

**Published:** 2012-06

**Authors:** Shan-qi Guo, Yi-zhuo Zhang

**Affiliations:** Department of Hematology, Tianjin Medical University Cancer Institute and Hospital, Key Laboratory of Cancer Prevention and Therapy of Tianjin, Tianjin 300060, China

**Keywords:** lymphoma, histone deacetylases, therapeutics, epigenomics

## Abstract

Lymphomas encompass a group of malignancies that originate in the lymph nodes or other lymphoid tissues. Epigenetic modification, especially by histone deacetylase (HDACs), plays a key role during the occurrence and development of lymphomas. Consequently, HDAC inhibitors (HDACIs), a class of gene expression-modulating drugs, have emerged as promising mechanism-based agents for the treatment of lymphomas. This review presents the rationale of HDAC inhibition, describes the epigenetic-based mechanisms of action of HDACIs, discusses their clinical efficiency, and summarizes the current and future developments in this field.

## Introduction

Apart from DNA methylation, the post-transcriptional modification of histone is another significant epigenetic process for regulating gene expression. Histone deacetylation is mediated by two groups of enzymes with opposite functions: histone acetyltransferases (HATs) and the histone deacetylases (HDACs). Both control the dynamic balance between the chromatin structure and gene expression of proteins involved in the regulation of a variety of functions, including cell survival, cell proliferation, angiogenesis, inflammation, and immunity. Given their intrinsic cytotoxic properties and combinatorial effects with other conventional therapies, HDAC inhibitors (HDACIs) show promising clinical utility in cancer treatment. The anti-tumor activity of HDACIs has been confirmed in a number of Phase I/II clinical trials. These trials indicate that HDACIs have certain effects on cutaneous T-cell lymphoma (CTCL), Hodgkin’s lymphoma (HL), myeloid tumors, and solid tumors ^[^^1^^]^. The HDACIs vorinostat and romidepsin have both been approved by the US Food and Drug Administration for the treatment of CTCL.

## HDACs

Histone deacetylation plays a part in signal transduction. Many cellular processes, including cell cycle control, apoptosis, protein degradation, angiogenesis, invasion, and cell motility, mainly depend on HATs and HDACs ^[^^2^^]^.

HDACs are major proteins that regulate diverse cellular functions by catalyzing the removal of acetyl groups from lysine residues in histone amino termini, leading to chromatin condensation and transcriptional repression. Conversely, HATs acetylate the ε-amino tails of lysine residues and neutralize the positive charge on histone tails. HATs also weaken the interaction between histones and negatively charged DNA, thus resulting in a more open, transcriptionally permissive chromatin structure that increases the accessibility for transcriptional processes ^[^^3^^]^. In addition to histones, numerous nonhistone proteins such as transcription factors (p53, STAT3, MYC, E2F, NF-κB, etc.), α-tubulin, and heat shock protein-90 can be regulated by HAT-mediated post-translational acetylation and HDAC-mediated deacetylation ^[^^4^^]^.

To date, 18 HDACs have been identified in humans. These HDACs are grouped into two major categories: zinc-dependent HDACs (Class I, II, and IV) and NAD-dependent HDACs (Class III). They are further divided into four major classes based on their homology to yeast HDACs, subunit localizations, and enzymatic activities. Class I HDACs (HDAC1, 2, 3, and 8) are homologous to the yeast RPD3 protein, can generally be detected in the nucleus, and show ubiquitous expression in various human cell lines and tissues. Class II HDACs (HDAC4, 5, 6, 7, 9, and 10) share homologies with yeast Hda1 protein. Class III HDACs (SIRT1, 2, 3, 4, 5, 6, and 7) are homologues of yeast protein Sir2 and require NAD^+^ for their activity to regulate gene expression in response to changes in the cellular redox status. Interestingly, based on its multiple functions, SIRT1 can function as a tumor promoter and suppressor by negatively regulating multiple pathways that include both tumor suppressors (p53 and FOXO) and oncogenic proteins (survivin, β-catenin, and NF-κB) ^[^^5^^]^. HDAC11 is the sole member of Class IV HDACs, which shares sequence similarity with the catalytic core regions of both the Class I and II enzymes ^[^^2^^]^.

Due to their fundamental roles in gene expression and diverse effects on histones as well as nonhistones, HDACs are considered as promising treatment targets for cancer, such as lymphomas ^[^^6^^]^. Gloghini et al. ^[^^7^^]^ demonstrated that the expression of HDAC6 is the most frequently altered among HDAC enzymes. HDAC6 is also confirmed to be consistently expressed at low levels in lymphoid cell lines, implying that selective therapies for lymphoma may exist.

## HDACIs

HDACIs are a group of chemically diverse compounds that inhibit the activities of HDACs. Several HDACIs are currently being evaluated for the treatment of some types of cancer ^[^^1^^]^.

Based on their chemical structures and enzymatic activities, HDACIs can be structurally grouped into 5 classes, namely, hydroximates, cyclic peptides, aliphatic acids, benzamides, and electrophilic ketones ^[^^8^^]^. Hydroxamic acids were the first to be discovered, and are thus the most extensively studied HDACIs with strong activities and simple structures. The members of this class, such as trichostatin A, vorinostat (SAHA), and panobinostat (LBH589), are potent unselective inhibitors of both Class I and II HDACs. The interaction between hydroxamic acids and zinc in the active site pockets of HDACs mainly occurs via a functional sulfydryl group. Aliphatic acids including butyric acid, benzene acid, valproic acid, and so on, which have relatively weak inhibitory activity, low bioavailability, and fast metabolism, making them the least attractive HDACIs. Cyclic peptides comprise both epoxyketone- and non-epoxy ketone-containing tetrapeptides. These peptides are the most structurally complex group of HDACIs, including depsipeptide (FK228), apicidin, trapoxin, and other compounds. Benzamides have lower activity than the corresponding hydroxamic acids and cyclic peptides. The compounds in this class contain a benzamide part and are selective, potent inhibitors of Class I HDACs. The representative compounds are MS-275, MGCD103, and CI-994 ^[^^9^^]^.

HDACIs exert a myriad of biological effects, including the induction of cell differentiation/apoptosis, cell-cycle arrest, and induction of autophagic cell death. HDACIs modify gene expression and alter the acetylation status of transcription factors (e.g., E2F, Stat3, p53, NF-κB, TFIIE, and Rb protein) and other proteins involved in transcription, eventually leading to cell death, DNA damage ^[^^10^^]^, and blocked activity of chaperone proteins.

### Effects on growth arrest and cell cycle control

HDACI-induced growth arrest is closely linked to the induction of p21 through the hyper-acetylation of chromatin at CDKN1A (p21^WAF1/CIP1^) promoter and the transcriptional activation of CDKN1A. Cell cycle arrest is linked to the increasing level of p21^WAF1/CIP1^, which results in the decreased expression of cyclin proteins and cyclin-dependent kinases^[^^11^^]^.

### Effects on apoptosis pathways and autophagy

HDACIs act mainly via the death receptor pathway (extrinsic) or mitochondrial pathway (intrinsic) to activate caspase and induce tumor cell apoptosis ^[^^12^^]^. The increasing levels of the death receptor ligands FasL and TRAIL caused by HDACIs are not observed in normal cells, suggesting the selectivity of HDACIs. These compounds can also activate the pro-apoptotic factors Bcl-2 family (Bax, Bak, Bim, and Bmf) while downregulating antiapoptotic proteins (Bcl-2, Bcl-XL, MCL-1, XIAP, and survivin) that have been associated with the resistance to HDACI-induced apoptosis. A recent study showed that vorinostat can increase the level of NOX, an enzyme promoting the generation of reactive oxygen species (ROS), indicating that ROS production may be a significant mechanism of cell death ^[^^13^^]^. Most reports consider that non-apoptotic cell death induced by HDACIs is related to autophagy, but the specific mechanism remains unclear ^[^^14^^]^.

## Clinical Application of HDACIs in the Treatment of Lymphoma

The rapid development in understanding the biology of lymphomas and accumulating evidence on the efficacy of HDACIs in treating lymphoid malignancies make HDACIs a class of efficient and promising therapeutic agents for lymphomas ^[^^15^^, ^^16^^]^. All currently available HDACIs mainly inhibit Class I and II HDACs. Many structurally diverse compounds can bind to HDACs and inhibit their enzymatic activity.

### HL

The cure rate of HL is over 80%, thus, it is considered one of the most curable human cancers. However, patients who relapse and are refractory, especially those who fail first- or second-line regimens, generally have poor prognosis and early mortality. Recent clinical trials have demonstrated that HDACIs have a promising effect on relapsed or refractory HL^[^^17^^]^.

### LBH589

LBH589 is a hydroxamate analog that mainly inhibits Class I, II, and IV HDACs with evidence of activity in myeloid malignancies and cutaneous T-cell lymphoma. LBH589 is one of the most potent HDACIs for HL *in vitro*. A phase IA/II multicenter study being conducted by Dickinson et al. ^[^^18^^]^enrolled 13 patients with HL, and computed tomography revealed that 5 of them (38%) achieved partial remission. Panobinostat appears to be well tolerated, and its most common side effect is thrombocytopenia. The maximum tolerated dose (MTD) was defined as 40 mg a day. The latest interim response rate of this ongoing study is 21% (17/81), indicating the promising clinical activity and good safety profile of panobinostat. Subsequently, based on experimental data, another phase II clinical trial administered panobinostat at the MTD to 61 relapsed or refractory HL patients. One patient achieved partial remission (PR), 10 achieved complete remission (CR), and 31 had stable disease ^[^^19^^]^. Thus, LBH589 has encouraging clinical activity in relapsed or refractory HL patients, with fewer side effects.

### Mocetinostat (MGCD0103)

A novel oral benzamide, mocetinostat selectively inhibits HDAC Classes I and IV, showing potent anti-proliferative activity in inhibiting Class I HDACs in HL cell lines. Mocetinostat can reportedly induce TNF-α, activate NF-κB, and regulate Jak/STAT signaling components to favor cell death, and downregulate the expression of CD30 receptor (cause of malignant Hodgkin and Reed-Sternberg cells) ^[^^20^^]^. The safety and efficacy of MGCD0103 given orally thrice per week (85 or 110 mg starting doses) were recently evaluated in a phase II clinical trial in patients with relapsed and refractory HL. Although the response rate of the 100 mg group was 35% (7/20), the intolerable toxicity led to dose reduction and therapy discontinuation. The PR in the 85 mg group was 30%, with the grade 3/4 toxicity (mainly fatigue) reduced to 20%. Therefore, mocetinostat, at 85 mg thrice weekly is an effective single agent for relapsed or refractory HL with a manageable safety profile ^[^^9^^, ^^17^^]^.

### B-cell non-HL (NHL)

B-cell NHLs, the most common lymphomas, are classified into aggressive subtypes and indolent diseases. Diffuse large B-cell lymphoma (DLBCL) and follicular lymphoma (FL) are common types of NHLs. Given that relapse is common in B-cell lymphomas, novel effective treatments are significantly needed ^[^^21^^]^. At present, there is no HDACI approved specifically for the treatment of B-cell lymphomas, but a strong rationale and clinical trials on some HDACIs have shown their potent activity against B-cell NHL ^[^^22^^]^.

### SAHA

A pan inhibitor of both Class I and II HDACs, vorinostat reportedly has clinically efficiency in treating B-cell lymphomas. Recently, a phase I study on oral vorinostat at 2 doses (100 or 200 mg twice daily for 14 consecutive days, followed by a 1 week rest interval in a 3 week cycle) enrolled 10 Japanese patients, including 4 with FL, 2 with mantle cell lymphoma (MCL), and 2 with DLBCL. The overall response rate was 40%; two CR and one PR occurred in FL patients, and one CR occurred in an MCL patient. The common related adverse events were anorexia, hyperlipidemia, albuminuria, fatigue, nausea, diarrhea, hypocalcemia, and abnormal hematology (thrombocytopenia, anemia, leukopenia). The hematologic adverse reactions are reversible and patients recover early from these events simply by resting ^[^^22^^, ^^23^^]^. The potential single-agent activity of vorinostat in FL or MCL is highlighted. In another phase II trial using vorinostat to treat relapsed DLBCL, only 1 of 18 patients (median age=66 years; number of median prior therapies=2) achieved CR, with more than 468 days of remission time. Thus, vorinostat, as a single agent, does not show the expected activity in the treatment of B-cell lymphoma despite its tolerable toxicity ^[^^24^^]^.

### Belinostat (PXD101)

Another hydroxamic acid derivative, belinostat is the only HDACI in clinical trials that can be administered via multiple potential routes. In a phase I clinical trial for advanced B-cell malignancy treatment, belinostat was administered at different doses (600, 900, and 1,000 mg/m^2^/d i.v.). Among 16 patients with relapsed or refractory B-cell lymphomas, 5 patients including 2 with DLBCL achieved stable disease. The adverse events reported were nausea, vomiting, fatigue, and diarrhea, and the MTD was determined to be 1,000 mg/m^2^/d on days 1-5 in a 21-day cycle. This dose can also be used in patients with hematological neoplasms. Data of another phase I clinical trial for recurrent refractory lymphoma also showed an acceptable safety profile and more important signs of clinical efficiency of oral belinostat in terms of stabilizing the disease. Particularly, clinical responses were reported in all patients with mantle cell lymphoma ^[^^9^^, ^^22^^, ^^25^^]^.

### T-cell NHL

T-cell NHL and NK-cell neoplasms account for about 12% of all NHLs and 15%-20% of aggressive lymphomas. The T-cell phenotype conferred a worse clinical outcome than its B-cell counterpart, so relapsed or refractory lymphoma is still a significant clinical problem to be solved.

### SAHA

A total of 33 patients with relapsed or refractory CTCL were enrolled in a phase II clinical trial of oral vorinostat at different dosages and schedules. The results showed that the overall response rate (ORR) was 24.4%, the medium time to response (mTTR) was 11.9 weeks, and the medium duration of response (mDOR) was 15.1 weeks. Consequently, a 400 mg/day dose was considered optimum with the best safety profile. The related dose-limited toxicities include gastrointestinal disorders, anorexia, dehydration, fatigue, and bone marrow depression ^[^^26^^]^. A subsequent phase II study showed evidence of the prolonged safety and clinical benefits of vorinostat in 74 relapsed or refractory CTCL patients, wherein the ORR was 29.7%, the mTTR was shorter at only 8 weeks, and the mDOR was more than 185 days ^[^^27^^]^. Compared with other drugs, vorinostat has the advantage of quick response time, good tolerance, and convenient oral administration. All these data suggest the approval of vorinostat by the FDA for the treatment of relapsed or refractory CTCL in 2006 ^[^^28^^, ^^29^^]^.

### Romidepsin (FK228)

A total of 71 patients with relapsed or refractory CTCL enrolled in a phase II study conducted by the National Cancer Institute were given intravenous romidepsin at a dose of 14 mg/m^2^. The results showed that the ORR was 34% with CR observed in 4 patients, the mTTR was 2 months, and the mDOR was 13.7 months. The related side effects included fatigue, anorexia, and hematological abnormalities ^[^^30^^]^. A multi-institutional phase II study of romidepsin in relapsed and refractory peripheral T-cell lymphoma (PTCL) reported both CR and PR in patients with PTCL. The ORR for these 36 patients was 31%, with 3 patients (8%) achieving CR and 8 patients (22%) achieving PR ^[^^31^^]^. Therefore, romidepsin has significant single-agent activity against PTCL. Romidepsin received an orphan drug designation from the FDA for the treatment of non-Hodgkin’s T-cell lymphomas, including CTCL and PTCL, and it was approved for the treatment of relapsed or refractory CTCL by the FDA in 2009 ^[^^22^^]^.

## Conclusion and Future Perspectives

As a new class of epigenetic-based agents with minimal effects on normal tissues, HDACIs present potent and promising activities for the treatment of malignant lymphomas. By changing the histone acetylation status, HDACIs regulate gene expression and modulate the chromatin structure. They also have higher selectivities to tumor cells than to normal cells than other chemotherapeutic agents. Recently, checkpoint kinase 1, a component of the G2 DNA damage checkpoint, has become a research hotspot because of its function in the resistance of normal cells to HDACIs (such as vorinostat, romidepsin, or entinostat) ^[^^32^^]^.

However, there are still numerous problems to be solved, such as their specific anti-cancer mechanisms, the relationship between the concentration and treatment duration of HDACIs, and the standard treatment for lymphomas with the best efficiency and manageable safety profiles. Given the short half-life and fast metabolism rate of HDACIs, the proper dosage, measures to improve the pharmacodynamic stability, and meaningful biomarkers for prognosis prediction are also needed.
